# Development of a model for marburgvirus based on severe-combined immunodeficiency mice

**DOI:** 10.1186/1743-422X-4-108

**Published:** 2007-10-25

**Authors:** Kelly L Warfield, Derron A Alves, Steven B Bradfute, Daniel K Reed, Sean VanTongeren, Warren V Kalina, Gene G Olinger, Sina Bavari

**Affiliations:** 1 United States Army Medical Research Institute of Infectious Diseases, Fort Detrick, Maryland, USA

## Abstract

The filoviruses, Ebola (EBOV) and Marburg (MARV), cause a lethal hemorrhagic fever. Human isolates of MARV are not lethal to immmunocompetent adult mice and, to date, there are no reports of a mouse-adapted MARV model. Previously, a uniformly lethal EBOV-Zaire mouse-adapted virus was developed by performing 9 sequential passages in progressively older mice (suckling to adult). Evaluation of this model identified many similarities between infection in mice and nonhuman primates, including viral tropism for antigen-presenting cells, high viral titers in the spleen and liver, and an equivalent mean time to death. Existence of the EBOV mouse model has increased our understanding of host responses to filovirus infections and likely has accelerated the development of countermeasures, as it is one of the only hemorrhagic fever viruses that has multiple candidate vaccines and therapeutics. Here, we demonstrate that serially passaging liver homogenates from MARV-infected severe combined immunodeficient (scid) mice was highly successful in reducing the time to death in scid mice from 50–70 days to 7–10 days after MARV-Ci67, -Musoke, or -Ravn challenge. We performed serial sampling studies to characterize the pathology of these scid mouse-adapted MARV strains. These scid mouse-adapted MARV models appear to have many similar properties as the MARV models previously developed in guinea pigs and nonhuman primates. Also, as shown here, the scid-adapted MARV mouse models can be used to evaluate the efficacy of candidate antiviral therapeutic molecules, such as phosphorodiamidate morpholino oligomers or antibodies.

## Background

The family *Filoviridae *consists of two genera called ebolavirus (EBOV) and marburgvirus (MARV) that are considered significant public health threats due to their very high morbidity and mortality rates (up to 90% case fatality rate), human-to-human transmissibility, and environmental stability. Due to these characteristics, and the fact that the filoviruses have a low infectious dose [<1 plaque-forming units (pfu)] and can be easily produced to >10^8 ^pfu/ml *in vitro *or *in vivo *[1-4], the filoviruses are classified as biosafety level (BSL)-4 agents and Category A biothreat agents by the Centers for Disease Control and Prevention [5,6]. Initial symptoms of filovirus infection include nonspecific clinical signs such as high fever, headache, myalgia, vomiting and diarrhea, followed by leukopenia, thrombocytopenia, lymphadenopathy, pharyngitis, edema, hepatitis, maculopapular rash, hemorrhage, and prostration with death generally occurring within 5–10 days of infection [1,7].

The first known filovirus outbreaks occurred in simultaneously in both Germany and Yugoslavia in 1967 when laboratory workers became infected from blood and tissues of MARV-infected African green monkeys imported from Uganda [8,9]. Subsequent MARV cases or outbreaks have occurred in South Africa, Zimbabwe, Kenya, Democratic Republic of Congo, and Angola with case fatality rates ranging from 20% in Germany in 1967 [8,9] to >90% in Angola during 2004–5 [10]. It is generally considered that transmission of the filoviruses requires direct contact with blood, body fluids, or tissues from an infected individual [11,12], although droplet and aerosol transmissions may also occur [13].

Human-derived Marburg viruses (isolates Musoke, Ravn, and Ci67) are not lethal to immmunocompetent adult mice. Previously, an Ebola Zaire mouse-adapted virus was developed by performing 9 sequential passages of Ebola Zaire '76 virus in suckling mice followed by two sequential plaque picks. The resulting virus was uniformly lethal to mice after intraperitoneal inoculation [14]. Pathologic evaluation of infected mice identified similarities and differences between this model [14,15] and infections in nonhuman primates [16,17]. Similarities include the tropism of the virus for monocytes/macrophages and high viral titers in the spleen and liver tissues after infection [reviewed in [18]]. The mean time to death of infected mice is approximately 5–10 days, which is similar to that observed in infected cynomolgus and rhesus macaques.

A viable lethal mouse model for Marburg virus is critical to the filovirus vaccine research program to understand the immune mechanisms that need to be induced, or avoided, by vaccination. Furthermore, a mouse model would speed the testing and evaluation of new Marburg therapeutic candidates. This effort is currently impeded due to limitations in the numbers of guinea pigs that can be evaluated at one time (based on BSL-4 space limitations, as well as physical demands on investigators and technicians) and the large amounts of compounds that must be synthesized or purified for testing in guinea pigs, which are 20–50× the size of mice. The purpose of this work was to select a marburgvirus that caused death within a similar timeframe as monkeys or humans (7–10 days) in severe combined immunodeficiency (scid) mice. To accomplish this goal, we repeatedly passaged the liver homogenates of MARV-infected scid mice and then recorded their time to death. Once we identified rapidly lethal mouse-adapted viruses, we characterized the models by immunology and pathology studies. These scid mouse-adapted viruses will be used to explore the virulence factors associated with marburgvirus infection. Furthermore, the scid models of MARV infection will be particularly useful for screening candidate therapeutics for their ability to directly diminish viral replication in the absence of adaptive immune responses.

## Methods

### Virus and cells

Human-derived (wild-type) and mouse-adapted MARV-Musoke, -Ravn, and -Ci67 virus stocks were propagated no more than three passages in Vero or VeroE6 cells. The human-derived (wild-type) and mouse-passaged MARV-Musoke, -Ravn, and -Ci67 plaques were counted by standard plaque assay on Vero cells [19]. MARV-infected cells and animals were handled under maximum containment in a BSL-4 laboratory at the United States Army Medical Research Institute of Infectious Diseases.

### Animals

BALB/c severe combined immunodeficient (scid) mice, aged 4 to 8 weeks, of both sexes, were obtained from National Cancer Institute, Frederick Cancer Research and Development Center (Frederick, MD). Mice were housed in microisolator cages and provided autoclaved water and chow *ad libitum*. Research was conducted in compliance with the Animal Welfare Act and other federal statutes and regulations relating to animals and experiments involving animals and adhered to principles stated in the *Guide for the Care and Use of Laboratory Animals*, National Research Council, 1996. The facility where this research was conducted is fully accredited by the Association for Assessment and Accreditation of Laboratory Animal Care International.

### Mouse adaptation

The general approach to adapt MARV to mice was based on virus passage in scid (BALB/c background) mice to avoid usage of suckling mice to develop a lethal mouse-adapted Marburg virus. The goal was to isolate the viral population that was capable of migrating to target tissues/organs (i.e., liver) at the earliest time point. Each group consisted of 10 mice that were inoculated intraperitoneally (IP) with 1000 pfu of Marburg virus (isolate Musoke, Ci67, or Ravn). Two mice were euthanized on day 7, the livers removed, pooled, and homogenized in 10 ml of PBS. The liver homogenates were blindly passed (200 μl IP) and used to infect new mice to evaluate lethality of the next virus passage. Lethality was monitored in the remaining eight mice of each passage. The supernatants of the liver homogenates from each passage were introduced onto Vero cells to determine the viral titers by plaque assay [19].

### Viral challenge with 'scid-adapted' MARV

For the characterization studies, scid mice were injected IP with ~1000 pfu of 'scid mouse-adapted' MARV-Musoke, passage (P)10; Ravn P(10); or Ci67, P(15) diluted in PBS. After challenge, mice were observed at least twice daily for illness and death and in some experiments, daily weights were determined for each infected group.

### Hematologic studies

For mice, blood samples were obtained under anesthesia by cardiac puncture. Viremia was assayed by traditional plaque assay [19]. Hematological, cytokine, and D-dimer levels, as well as liver-associated enzymes, were measured as previously described [20,21].

### Pathologic sampling

Four animals from each group were randomly chosen for euthanasia on 2, 4, 6, and 8 days postchallenge for gross necropsy. A full complement of tissues from each mouse was fixed in 10% neutral buffered formalin and held in the BSL-4 laboratory for >21 days. The tissues were embedded in paraffin, sectioned for histology, and stained with hematoxylin and eosin for routine light microscopy or were stained by an immunoperoxidase method (Envision System – DAKO Corporation, Carpinteria, CA), using a mixture of two mouse monoclonal antibodies against MARV nucleoprotein (NP) and glycoprotein, or by the TUNEL method to detect apoptotic cells within the tissue samples.

### Adminstration of antisense PMO and filovirus-specific antisera

Two groups of 10 scid mice were each administered 1 ml of convalescent sera from guinea pigs that had survived either EBOV or MARV infection. The antibodies were administered IP 1 h after challenge. Both pools of antisera had 80% plaque reduction-neutralization titers of >1:160 against the homologous virus, but <1:20 against the heterologous virus. Alternately, another group of 10 scid mice were administered IP with 1 mg of a mixture of four MARV-specific phosphodiamidate morpholino oligomers (PMOs) targeting the AUG start site of VP24, VP35, NP, and L (kind gift of Dr. P.L. Iversen of AVI BioPharma, Inc., Corvallis, OR) 1 h after challenge. A control group received saline (i.e., vehicle) alone. The mice were challenged with 1000 pfu of 'scid-adapted' MARV-Ci67 and monitored for survival.

## Results

### Adaptation of Marburg viruses to scid mice

**Figure 1:**
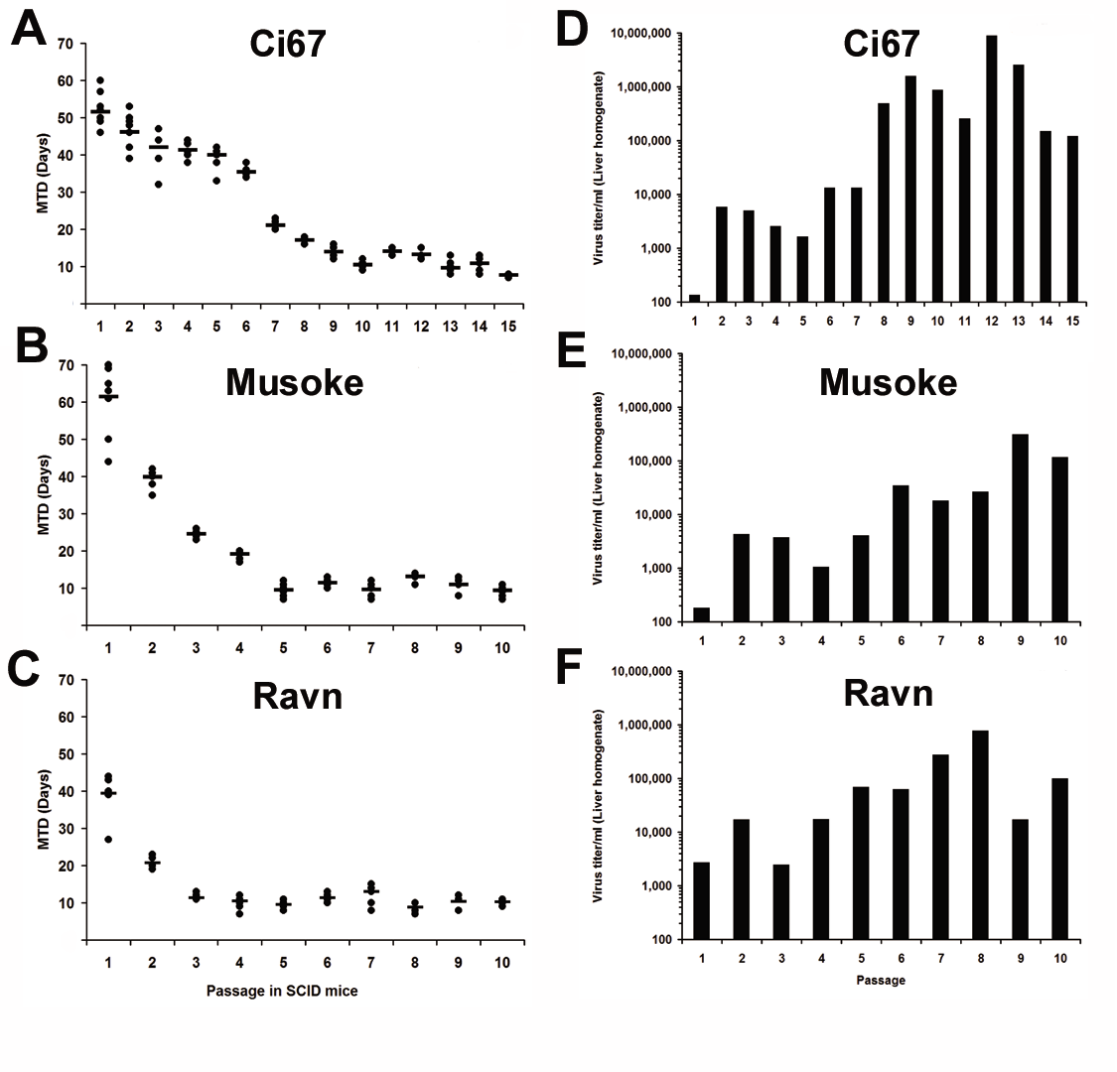
**Adaptation of MARV to severe combined immunodeficiency (scid) mice**. Groups of scid mice (n = 10) were infected with ~1000 pfu of wild-type MARV-Ci67, -Musoke, or -Ravn. The livers of two mice from each group were harvested 7–8 days after infection, pooled together, homogenized, and blind-passaged into a new group of naïve scid mice. Blind passaging proceeded until a mean time-to-death of 7–10 days was observed consistently through several passages. **(A-C) **The remaining eight mice from each group were monitored for survival and the data are presented as the time-to-death for each animal (filled circles) and the mean time-to-death (line). **(D-F) **The viral titers in the pooled liver homogenates were determined after each passage in scid mice. P15: Passage 15, P10: Passage 10.

Previously, an Ebola Zaire mouse-adapted virus was developed by performing 9 sequential passages of Ebola Zaire '76 virus in suckling mice [14]. We chose to take a slightly different approach, by repeatedly passaging MARV-Ci67, -Musoke, or -Ravn in scid mice after initial inoculation with the wild-type (i.e. human-derived) viruses. The livers of two mice were harvested on day 7–8 after each infection, pooled together, homogenized, and blind-passaged into naïve scid mice until a mean time-to-death (MTD) of ≤10 days was observed through several passages (Figure 1A–C). The starting time to death of the scid mice varied after injection with the wild-type MARV isolates. MARV-Musoke began with the highest MTD of 61.5 (± 9.67) days and dropped to 9.375 (± 1.30) days within 10 passages. For MARV-Ci67, the MTD was 51.6 (± 4.98) days for the wild-type virus and was 7.75 (± 0.46) days after 15 passages. The MTD for MARV-Ravn began at 39.4 (± 5.48) days and was 10.3 (± 0.71) days after 10 passages in scid mice. The viral titers in the liver homogenates from each passage were determined using plaque assay and we found an upward trend in the viral titers amongst the liver homogenates with increasing passage in mice (Figure 1D–F). The increase in viral titer in the day 7 liver homogenates seemed to correspond with a decrease in the MTD of the inoculated mice.

### Characterization of the 'scid-adapted' MARV mouse models

We next intended to characterize the rapidly lethal 'scid-adapted' mouse models of MARV-Musoke, -Ravn, and -Ci67 via serial sampling studies of infected scid mice. It was of particular interest to determine if the infection caused similar laboratory, immunological, and pathological responses in mice, as was observed in MARV-infected guinea pigs and nonhuman primates. Within 3–4 days after injection with the 'scid-adapted' MARV strains, mice developed anorexia, a hunched appearance, and exhibited decreased grooming. Some mice also appeared to have blood in their urine and many mice developed hind-limb paralysis after 'scid-adapted' MARV infection (data not shown).

**Figure 2:**
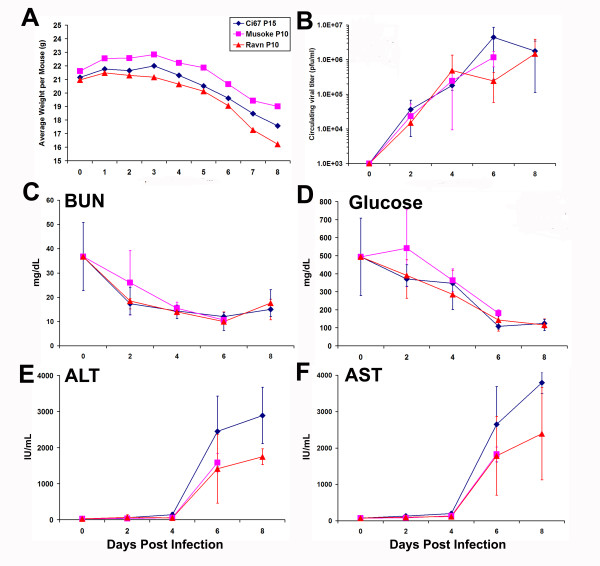
**Weight loss, viremia and serum chemistry values of mice infected with 'scid-adapted' MARV**. Scid mice were infected with 1000 pfu of the indicated 'scid-adapted' MARV (Ci67 P15, Musoke P10 or Ravn P10). **(A) **The weight of groups of 10 mice was assessed daily after infection with the 'scid-adapted' MARV. The data are expressed as the average weight of the mice in each group. **(B) **Viral titers were measured using standard plaque assay on serum samples obtained from terminal cardiac punctures of infected mice on 0, 2, 4, 6 or 8 days postinfection. Levels of **(C) **Blood urea nitrogen (BUN), **(D) **glucose **(E) **alanine transaminase (ALT), and **(F) **aspartate transaminase (AST) were measured at the indicated timepoints using serum collected by terminal cardiac puncture. Data for panels B-F are expressed as the average of values from four to five mice/timepoint and error bars indicate the standard deviation.

**Figure 3:**
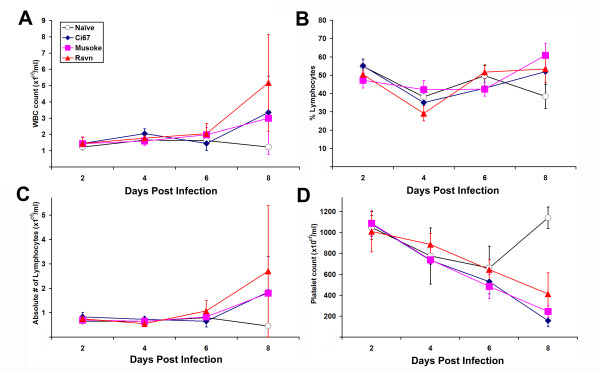
**Hematologic changes in mice infected with 'scid-adapted' MARV**. Scid mice were infected with 1000 pfu of Ci67 P15, Musoke P10 or Ravn P10 'scid-adapted' MARV or left uninfected (naïve). Whole blood was collected from individual mice (n = 4–5/timepoint) in EDTA via terminal cardiac puncture at the indicated timepoints. **(A) **Total numbers white blood cells (WBC), **(*B*) **percentage of lymphocytes, **(C) **absolute numbers of lymphocytes, and **(*D*) **platelet counts in the blood were assessed and are presented as the mean value (± standard deviation).

As expected, there was a noticeable and steady weight loss in mice infected with the 'scid-adapted' MARV beginning around 4–5 days after infection (Figure 2A). Similar to what is seen in guinea pigs and monkeys, infection with the 'scid-adapted' MARV viruses caused detectable viremia within 2 days of infection (Figure 2B). The viremia in all the mice increased logarithmically over the course of the infection and peaked around 10^6 ^pfu/ml in the serum at days 6–8 (Figure 2B). Serum levels of blood urea nitrogen (BUN) and glucose dropped sharply over time after infection of the scid mice (Figure 2C–D). As is seen in all other models of filovirus infection, indicators of liver health such as alanine transaminase (ALT) and aspartate transaminase (AST) function increased as the MARV disease progressed (Figure 2E–F). As shown by the total number of circulating white blood cells (WBC), percentage of lymphocytes, and absolute numbers of lymphocytes within the blood of the 'scid-adapted' MARV-infected mice, the very low number of circulating WBC and lymphocytes remained fairly steady until very late in the disease (Figure 3A–C). Most of the cells in the lymphoid system of scid mice are NK cells, except for a few immature B or T cells due to 'leakiness' of the scid system, and this explains the low WBC and lymphocyte counts in Figure 3A–C. A steady decrease in the number of platelets in the blood after infection was observed of the scid mice with the 'scid-adapted' MARV (Figure 3D). As would be expected with a coagulopathic disease and similar to filovirus infections in nonhuman primates [20,22], we observed elevations in serum d-dimer levels by ELISA with values >500 ng/ml by 6–8 days post infection (data not shown).

### Pathology characterization of the 'scid-adapted' MARV mouse models

**Figure 4:**
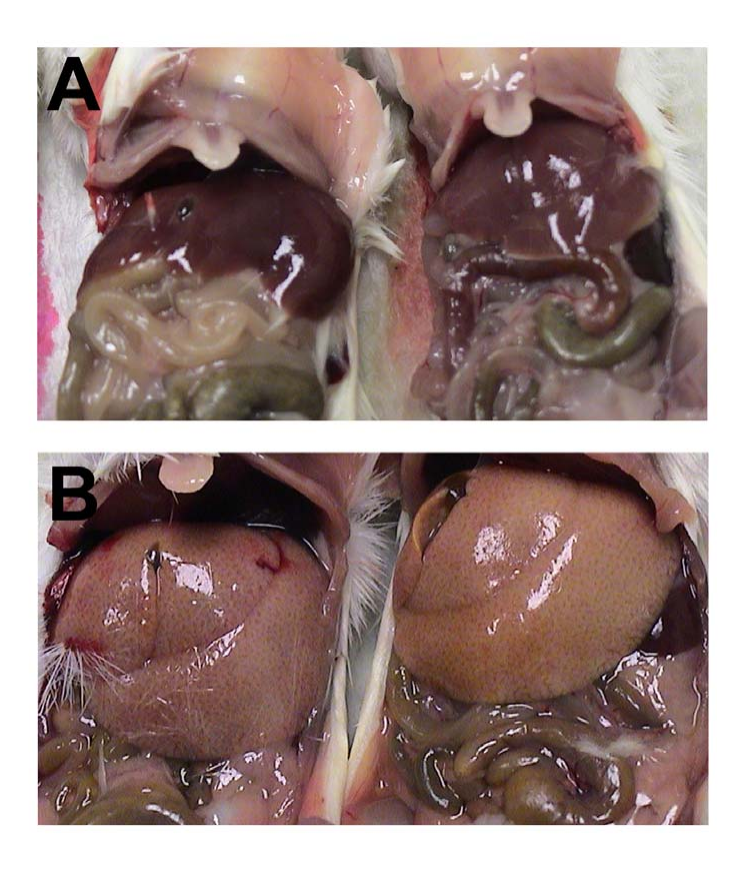
**Gross liver abnormalities upon necropsy of scid mice infected with 'scid-adapted' MARV**. **(A) **Livers of uninfected scid mice appear normal at the time of necropsy. **(B) **The livers from MARV-Ci67-infected scid mice were diffusely enlarged with rounded edges filling up to one-third of the abdominal cavity and mildly displacing abdominal organs. Additionally, the livers had become distinctively pale with an accentuated reticulated pattern.

Besides the noticeable and steady weight loss observed beginning around 4–5 days after infection, the most obvious and consistent gross necropsy finding in mice infected with the "scid-adapted" MARV occurred in the liver. When compared to uninfected scid mice (Figure 4A), livers from MARV-infected scid mice were diffusely enlarged with rounded edges filling up to one-third of the abdominal cavity and mildly displacing abdominal organs (Figure 4B). Furthermore, the livers had become diffusely yellowish-tan with an accentuated reticulated pattern and were extremely friable. Also consistently noted was that the blood of the 'scid-adapted' MARV-infected mice failed to clot post-mortem. To further characterize the lethal mouse models of MARV-Musoke, -Ravn, and -Ci67, histological analysis was performed on tissues from scid mice at 0, 2, 4, 6 and 8 days after infection. Histological lesions were mainly limited to the lymphoid organs and the liver.

**Figure 5:**
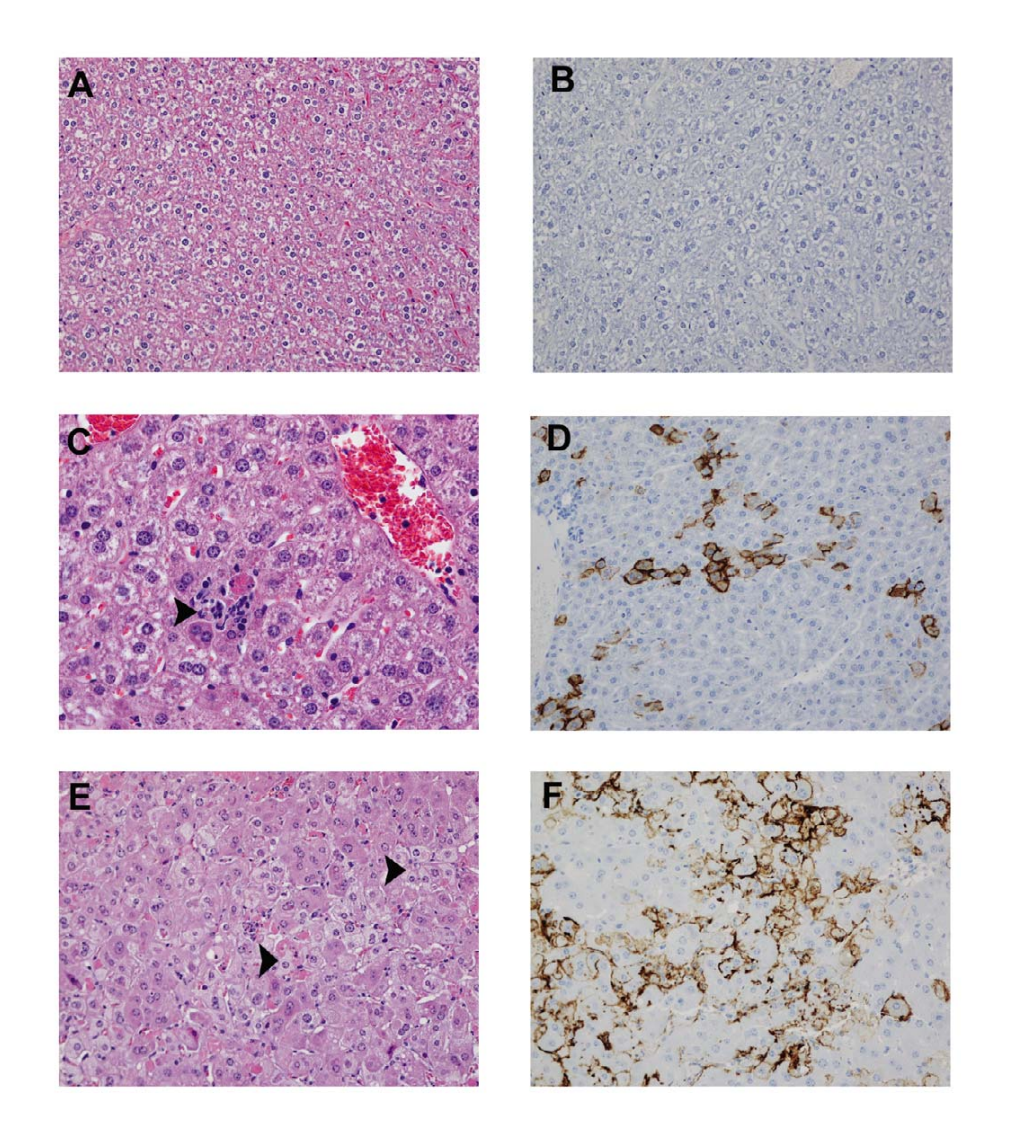
**Histological changes in livers of mice infected with 'scid-adapted' MARV**. Scid mice were challenged IP with 1000 pfu of 'scid-adapted' MARVCi67 and tissue samples were collected on days 0, 2, 4, 6, and 8 after challenge (n = 4–5/group). **(A, C, and E) **Tissues from the MARV-infected mice were stained with hematoxylin and eosin and representative pictures from day 0 **(A)**, 4 **(C)**, and 6 **(E) **are shown. The liver from the MARV-infected mouse contains multifocal necrosis, hepatocellular disruption, fatty cell degeneration, scattered hepatocellular viral inclusions, and inflammation composed of variable numbers of neutrophils and lesser numbers of macrophages and lymphocytes. **(B, D, and F) **Immunohistochemistry was performed on tissue sections from days 0 **(B)**, 4 **(D)**, and 6 **(F) **and MARV antigen appears brown. In the liver, MARV antigen is localized at the hepatocellular surface and most prominently noted along the sinusoids. Magnifications for **A-B **and **D-F **were 20× and panel **C **is shown at 40×.

Compared to uninfected scid mice (Figure 5A–B), within the livers of mice infected with "scid-adapted" MARV, there was single-cell hepatocellular necrosis with neutrophilic infiltrates beginning at day 4 which progressed from multifocal to coalescing areas of moderate to severe hepatocellular degeneration and necrosis (Figures 5C and 5E) by days 6 and 8. Fatty cell degeneration of the remaining hepatocytes was also a consistent finding at days 6 and 8. TUNEL-positive apoptotic-like bodies were frequently co-located within areas of hepatocellular necrosis and foci of neutrophilic inflammation (data not shown). Immunohistochemically, within 4 days of infection, many hepatocytes and Kupffer cells expressed strong surface immunoreactivity for MARV antigen (Figure 5D) and within 6 days, almost all hepatocytes and Kupffer cells were positive for MARV antigen.

**Figure 6:**
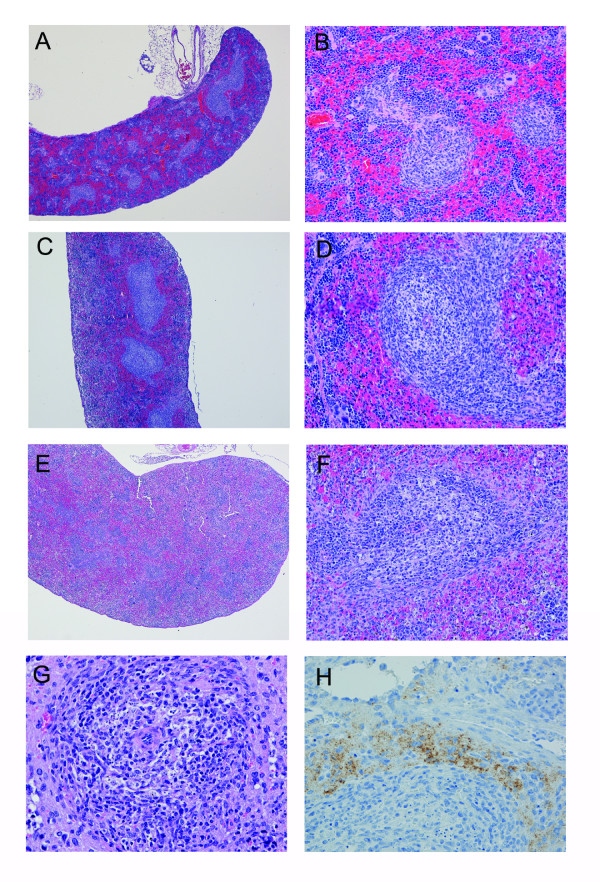
**Histological changes in spleens of mice infected with 'scid-adapted' MARV**. Scid mice were challenged IP with 1000 pfu of 'scid-adapted' MARV-Ci67 and tissue samples were collected on days 0, 2, 4, 6, and 8 after challenge (n = 4–5/group). **(A-G) **Tissues from the MARV-infected mice were stained with hematoxylin and eosin and representative pictures from day 0 **(A-B)**, 4 **(C-D)**, and 6 **(E-G) **are shown. (***A-B***) Control scid mouse sampled at day 0 (i.e. uninfected) contain abnormal spleen morphology due to lack of B and T lymphocytes. **(C-F) **Spleens from the MARV-infected scid mice at days 4 and 6 display increasingly more visual loss of cells in both the red and white pulp. **(G) **At late stages of the disease, the spleen contains notable necrosis/apoptosis of lymphocytes often with tingible body macrophages and large lymphoblasts in the white pulp. **(H) **Immunoperoxidase stain of a spleen from a scid mouse at 6 days postinfection showing presence of Marburg viral antigen (brown). Magnifications were 4× for panels **A, C**, and **E**, 20× for panels **B**, **D**, and **F**, and 40× for panels **G-H**.

**Figure 7:**
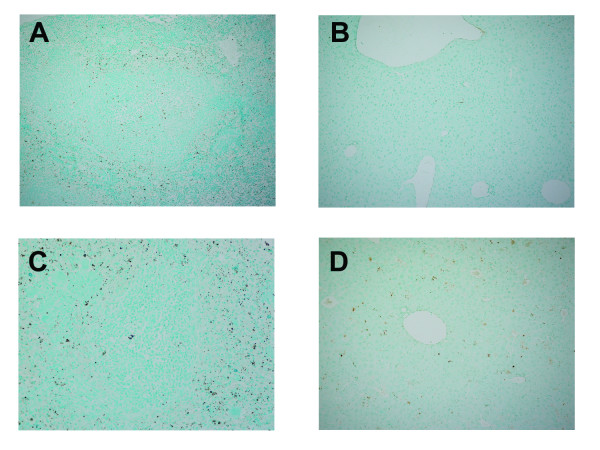
**Apoptosis within the spleen and liver of 'scid-adapted' MARV-infected mice**. Sections of the spleen and liver from mice infected with 'scid-adapted' MARV-Ci67 were stained using a TUNEL assay. **(A-B) **Control scid mouse sampled at day 0 (i.e. uninfected) contain TUNEL-positive cells, indicated by brown staining, in the spleen (A) and liver (B) due to natural turnover of naïve 'break-through' lymphocytes. **(C-D) **Increased number of TUNEL-positive cells in the spleen (C) and liver (D) of MARV-infected scid mice at day 6 postinfection.

As compared to the spleens of uninfected mice (Figures 6A–B), there was multifocal lymphocyte depletion and lymphocytolysis in the periarteriolar lymphoid sheaths (PALS) and follicles of the MARV-infected scid mice (Figures 6C–F). These changes were minimal to mild at 4 days postinfection, but more severe by day 6 postinfection. Much of this lymphocyte damage appeared due to apoptosis of cells within the red and white pulp based on TUNEL staining of tissues (Figure 7). We observed increased numbers of apoptotic-like bodies labeled by TUNEL as early as days 2 and 4 postinfection, with greater numbers of TUNEL-positive bodies at days 6 and 8 postinfection. In mice killed at 6 or 8 days postinfection, the spleens of infected mice contained large, lymphoblastic cells within splenic marginal zones (Figure 6G). Consistent with previous studies in other filovirus animal models [14-16], the majority of the MARV-infected cells within the spleen were located within the red pulp and appeared to be phagocytic cells such as macrophages and dendritic cells (Figure 6H).

**Figure 8:**
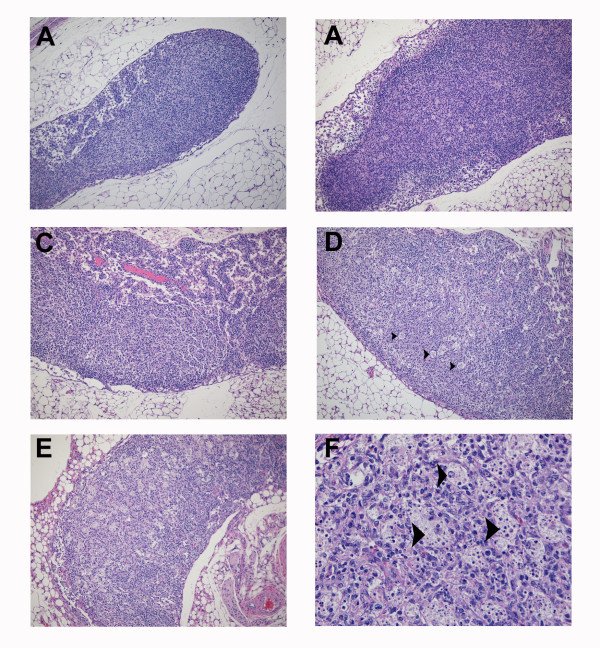
**Progression of histologic lesions within the mesenteric lymph nodes of mice infected with "scid-adapted" MARV**. Scid mice were challenged intraperitoneally with 1000 plaque-forming units of 'scid-adapted' MARV-Ci67 and tissue samples were collected on days 0, 2, 4, 6, and 8 after challenge (n = 4–5/group). **(A-F) **Tissues from the MARV-infected mice were stained with hematoxylin and eosin and representative pictures from day 0 **(A)**, day 2 **(B)**, day 4 **(C)**, and days 6 and 8 **(D, E, F) **are shown. **(A) **Control scid mouse sampled at day 0 (i.e. uninfected) contain abnormal lymph node morphology due to a paucity of B and T lymphocytes and failure of follicle development. **(B) **No significant histologic changes compared to uninfected lymph nodes observed at day 0. **(C) **By day 4, mesenteric lymph nodes from the MARV-infected scid mice exhibited minimal to mild lymphoid depletion and a slight increase in the number of tingible body macrophages. (**D, E**) At days 6 and 8, lymphoid depletion and lymphocytolysis was a consistent finding in the mesenteric lymph nodes of all MARV-infected scid mice. (**F**) At day 8, note the increased numbers of tingible body macrophages containing variably sized apoptotic-like bodies. Magnifications were 10× for panels **A, B, C, D**, and **E **and 40× for panel **F**. Tingible body macrophages are indicated by arrow heads.

Although no histologic changes were observed in the mesenteric lymph nodes at day 2 as compared to lymph nodes of uninfected mice (Figure 8A–B), cells labeled for Marburg virus antigen were occasionally present in medullary cords, surrounding high endothethelial vessels, and in the subcapsular sinuses at this timepoint (data not shown). Low to moderate numbers of virus-labeled histiocytes were present in the subcapsular, cortical, and medullary sinuses and parafollicular cells at days 4 and 6 postinfection. By day 4, there was minimal to mild lymphoid depletion and a slight increase in the number of tingible body macrophages in the mesenteric lymph nodes of all mice examined (Figure 8C). At days 6 and 8, moderate lymphoid depletion and lymphocytolysis were present in all mesenteric lymph nodes (Figure 8D–F).

Significant histologic or immunohistochemical findings attributed to "scid-adapted" MARV were not noted in any other tissues examined except the thymus and adrenal glands. Rarely, few MARV infected medullary cells interpreted as either thymic macrophages or dendritic interdigitating cells were observed at day 4. Additionally, MARV antigen was observed in few scattered cortical cells of the zona fasciculata and zona reticularis at days 6 and 8.

### Use of the 'scid-adapted' MARV model to assess the efficacy of potential therapeutics for MARV

**Figure 9:**
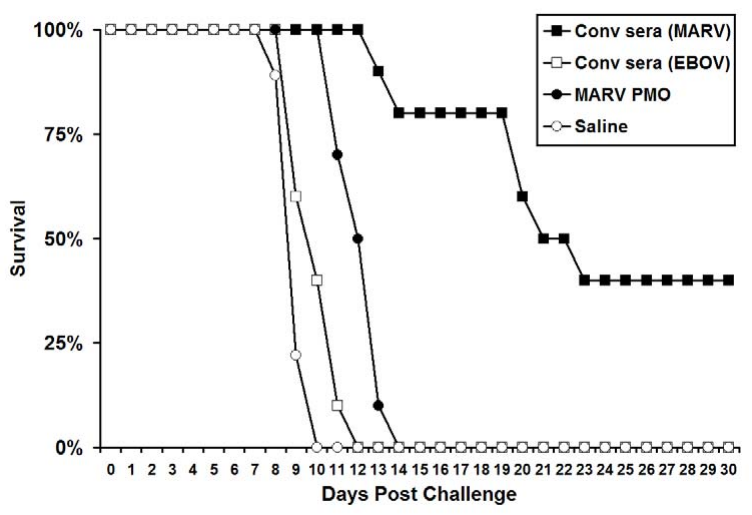
**Use of the 'scid-adapted' MARV model to assess the efficacy of potential therapeutics for MARV**. Scid mice were infected IP with ~1000 pfu of 'scid-adapted' MARV-Ci67. At 1 h postchallenge, groups of scid mice (n = 10) were treated IP with 1 ml of convalescent serum from guinea pigs that had survived MARV or EBOV challenge. Alternately, groups of mice were treated with 1 mg each of VP24, VP35, NP and L PMOs or saline alone as a vehicle control. The mice were monitored for >70 days for survival and the data are presented on a Meier-Kaplan curve as percent survival for each group.

To demonstrate the utility of our recently developed and characterized 'scid-adapted' MARV (Ci67, Musoke, and Ravn) in screening potential anti-MARV therapeutics, we treated scid mice that were infected with 'scid-adapted' MARV-Ci67. Since the scid mice do not have functional B or T cells and cannot mount an adaptive response to clear the virus, we only expected to see a delay in the mean time-to-death and not a survival benefit in these experiments. In the first portion of this experiment, 1 ml of pooled sera from convalescent guinea pigs that were previously infected with EBOV-Zaire or MARV-Musoke was administered IP 1 h after challenge to the MARV (Ci67)-infected scid mice. The scid mice that were treated with MARV convalescent sera had a MTD of >23 days (Figure 9). This was greatly increased when compared to the scid mice that had been treated with sera from EBOV convalescent guinea pigs (MTD = 12 days, P value < 0.001). Additionally, 40% of scid mice receiving anti-MARV sera survived until euthanasia at >70 days post infection with scid-adapted MARV-Ci67. In the second portion of this experiment, we tested the efficacy of a combination of four anti-MARV PMOs targeting VP24, VP35, NP and L (Figure 9). Scid mice that received the combination of anti-MARV PMO molecules at 1 h postinfection with 'scid-adapted' MARV-Ci67 had a significant delay in their mean time to death of 14 days, as compared to those receiving only saline (MTD = 9 days, P value < 0.001). Because transfer of antibody [23,24] or treatment with anti-MARV PMOs [Warfield *et al*., unpublished data] can protect guinea pigs, we feel that the delay to death observed in this model is an important indicator of anti-viral activity of these potential MARV treatments.

## Discussion

In previous studies, scid mice became ill and died within 3–4 weeks after inoculation with ZEBOV ('76), Sudan EBOV, or GP-adapted MARV-Ravn, but not with the other viruses [25]. However, the scid mice in these studies were only observed for 40 days after the infection – a much shorter time than we found required to produce lethal disease with the human-derived, wild-type viruses. The MTD of scid mice infected with the wild-type MARV isolates was not previously reported elsewhere. We found the time-to-death using wild-type MARV infections in scid mice much too long (50–70 days) to feasibly screen the efficacy of a large number of potential therapeutics *in vivo*. Therefore, we passaged the viruses until the time to death was consistently in the range of 7–10 days. These more virulent 'scid-adapted' viruses will allow for more rapid and efficient testing of candidate prophylactic and therapeutic treatments against multiple MARV isolates.

Initial serial sampling studies to characterize the pathology of these more virulent, scid-adapted MARV strains indicate similarities to the filovirus disease observed in other models. After parenteral challenge, the incubation period for MARV is 2 to 6 days, with death typically occurring between 7 and 11 days after infection in both guinea pigs and nonhuman primates [26-30]. Initial indicators of MARV disease in all the animal models include fever, anorexia, rash, huddling, weight loss, dehydration, and diarrhea. More severe complications such as prostration, failure to respond to stimulation, hind limb paralysis, and bleeding from injection sites and/or body orifices develop at later times after infection (i.e., 6–10 days) [26-30]. As noted here and in other models, the liver and spleen are tissues most consistently affected by MARV, as assessed by gross appearance, microscopy and histology. Based on pathology studies of the scid mice, guinea pigs, and nonhuman primates, the primary targets of MARV infection appear to be phagocytic cells, followed by hepatocytes, endothelial cells and fibroblastic cells [26-30]. Clinically, the scid mouse model appears to also be similar to the guinea pig and nonhuman primate models. MARV virus was present at increasingly high titers in the blood (Figure 2A), liver, spleen, kidneys, and other major organs (data not shown). Furthermore, early hematological and immunological changes included lymphopenia, variable neutrophilia, and profound thrombocytopenia [Figure 4 and [26-30]]. Notable alterations in serum chemistry levels, especially liver enzymes, occurred with increasing severity after infection (Figure 3). However, unlike nonhuman primates, rodents such as mice, guinea pigs, and hamsters are not susceptible to primary human isolates of MARV virus directly from blood or organ homogenates derived from infected patients [27,29-31].

Rodents infected with filoviruses appear to have slightly different coagulopathic responses than filovirus-infected nonhuman primates [14,26-30,32]. Similarities of the models include profound and rapid loss of circulating platelets, increased D-dimer levels, and uncontrolled bleeding (Figure 3D, data not shown, and [32]). For EBOV, rodents do not display all the characteristics of disseminated intravascular coagulation (DIC) that filovirus-infected nonhuman primates show including prolongation of PT and aPTT, circulating fibrin degredation products (FDPs), decreased plasma fibrinogen, and increased tissue fibrin deposition [32]. Not all these parameters have yet been tested for the MARV scid mouse model and will surely be the subject of future work.

Sequence comparisons of the original wild-type and more virulent scid-adapted MARV are required. Based on previous reports with mouse and guinea pig-adapted EBOV [18,33,34], we predict changes in VP24, VP35, NP, and L are likely to be important for enhanced virulence of the 'scid-adapted' MARV. VP24 was recently implicated in host pathogenicity as VP24 is an interferon antagonist that functions by binding karyopherin-α1 and blocking nuclear accumulation of the interferon signaling molecule stat1 [35,36]. The NP, VP35, and L proteins are all critical for viral replication and alterations in these proteins may lead to advantages in viral replication/growth within a given host species. NP is the viral nucleoprotein that tightly couples with the viral RNA [37]. Together, the L protein, VP30, and VP35 form the filovirus RNA-dependent RNA polymerase [37]. The VP35 is also implicated in blocking interferon (IFN) type-I responses in filovirus-infected cells by inhibiting double-stranded RNA-mediated activation of interferon regulatory factor 3, a transcription factor which triggers expression of interferon and interferon-stimulated genes [38-41]. Future experiments using reverse genetics could help demonstrate which of the acquired mutations were important for adaptation to mice.

This scid mouse model of MARV infection has obvious uses as a model for analysis of therapeutics and candidate antibodies. It will be much more efficient for the purposes of quickly screening lead compounds and neutralizing antibodies than guinea pigs or nonhuman primates. Before the development of this novel scid mouse model of MARV, a large quantity of antiviral compound and/or antibodies was required to achieve relevant physiological levels in guinea pigs, which are 20–50 times larger than mice, for the purposes of initial *in vivo *efficacy studies. Furthermore, guinea pigs require much larger cages, limiting the number of animals within a study and are also much more difficult and dangerous to handle under BSL-4 conditions than mice, requiring at least two laboratory personnel for treatments and challenges. A delay in time to death in this newly developed scid mouse model of MARV infection will indicate a positive result that should be followed up in the more intensive and expensive guinea pig studies. Thus, this novel MARV mouse model will allow for faster and more efficient *in vivo *screening of potential MARV prophylactics and therapeutics.
